# Increased Sestrin3 Contributes to Post-ischemic Seizures in the Diabetic Condition

**DOI:** 10.3389/fnins.2020.591207

**Published:** 2021-01-15

**Authors:** Zhongshan Shi, Zhigang Lei, Fan Wu, Luoxing Xia, Yiwen Ruan, Zao C. Xu

**Affiliations:** ^1^Department of Anatomy and Cell Biology, Indiana University School of Medicine, Indianapolis, IN, United States; ^2^Guangdong-Hongkong-Macau Institute for CNS Regeneration, Jinan University, Guangzhou, China; ^3^Department of Neurology, Sun Yat-sen Memorial Hospital, Sun Yat-sen University, Guangzhou, China; ^4^Third Affiliated Hospital of Guangzhou Medical University, Guangzhou, China; ^5^Jiangsu Province Co-innovation Center of Neuroregeneration, Nantong University, Nantong, China

**Keywords:** epilepsy, stroke, hyperglycemia, excitability, SESN3

## Abstract

Seizures are among the most common neurological sequelae of stroke, and diabetes notably increases the incidence of post-ischemic seizures. Recent studies have indicated that Sestrin3 (SESN3) is a regulator of a proconvulsant gene network in human epileptic hippocampus. But the association of SESN3 and post-ischemic seizures in diabetes remains unclear. The present study aimed to reveal the involvement of SESN3 in seizures following transient cerebral ischemia in diabetes. Diabetes was induced in adult male mice and rats via intraperitoneal injection of streptozotocin (STZ). Forebrain ischemia (15 min) was induced by bilateral common carotid artery occlusion, the 2-vessel occlusion (2VO) in mice and 4-vessel occlusion (4VO) in rats. Our results showed that 59% of the diabetic wild-type mice developed seizures after ischemia while no seizures were observed in non-diabetic mice. Although no apparent cell death was detected in the hippocampus of seizure mice within 24 h after the ischemic insult, the expression of SESN3 was significantly increased in seizure diabetic mice after ischemia. The post-ischemic seizure incidence significantly decreased in SESN3 knockout mice. Furthermore, all diabetic rats suffered from post-ischemic seizures and non-diabetic rats have no seizures. Electrophysiological recording showed an increased excitatory synaptic transmission and intrinsic membrane excitability in dentate granule cells of the rat hippocampus, together with decreased *I*_*A*_ currents and Kv4.2 expression levels. The above results suggest that SESN3 up-regulation may contribute to neuronal hyperexcitability and seizure generation in diabetic animals after ischemia. Further studies are needed to explore the molecular mechanism of SESN3 in seizure generation after ischemia in diabetic conditions.

## Introduction

Stroke is the major cause of death in the world. Seizures/epilepsy is a common complication after stroke (Bladin et al., 2000; Li et al., 2010). It has been reported by the American Diabetes Association that diabetic patients have a 2–4 fold higher risk of inducing a stroke. Hyperglycemia in people with diabetes will not only damage the central nervous system, but also increase the possibility of seizures following stroke (Ottman et al., 2011). Therefore, to reveal the molecular mechanism underlying post-ischemic epilepsy in diabetic animals may bring out a new clue in treatment of these patients.

Sestrins are a family of stress-inducible proteins, consisting of Sestrin1 (SESN1), Sestrin2 (SESN2) and Sestrin3 (SESN3). Sestrin-mediated inhibition of mTORC1 occurs via AMPK-mediated activation of the TSC1/2 complex (Budanov and Karin, 2008). The Sesn1 and Sesn2 genes are under transcriptional control of p53, while SESN3 is transcriptionally regulated by the AKT/FOXO axis, through FOXO1/FOXO3a-mediated gene expression (Budanov et al., 2004, 2010). SESN3 is a strong genetic risk factor that regulates proconvulsive cytokines and genes (Johnson et al., 2015) and plays a key role in metabolic homeostasis (Lee et al., 2012). It has reported that SESN3 regulates human hippocampal epilepsy (Johnson et al., 2015). However, the role of SESN3 in diabetes after stress, such post-ischemia seizures, remains unclear.

Hippocampus is a critical structure as an epileptic focus and epileptogenic zone. Increase of neuronal excitability is a widely recognized cause of seizures/epilepsy generation. It is conceivable that both membrane excitability and excitatory synaptic transmission are enhanced in hippocampal neurons in diabetic animals after ischemia, which results in seizures and epilepsy (Luhmann and Heinemann, 1992; Congar et al., 1995). In epileptic animals, inhibitory basket cells in the dentate gyrus receive less excitatory synaptic drive, and the transmission failure at basket cell-to- granule cell is increased (Zhang and Buckmaster, 2009). A-type potassium currents (*I*_*A*_ currents) are voltage-dependent, transient outward K^+^ currents that quickly activate at subthreshold membrane potentials and rapidly inactivate. Considerable evidence has shown that *I*_*A*_ currents play a pivotal role in the regulation of neuronal excitability (Carrasquillo et al., 2012). It has been reported that Kv4.2 knockout mice increased susceptibility to convulsant stimulation and Kv4.2 truncation mutation was seen in a patient with temporal lobe epilepsy (Singh et al., 2006; Barnwell et al., 2009). Our previous study has shown that traumatic brain injury (TBI) causes a downregulation of *I*_*A*_ in hippocampal neurons, which is associated with the hyperexcitability in the post-traumatic hippocampus and leads to an enhanced seizure susceptibility (Lei et al., 2012). And the reduction of *I*_*A*_ channels in hippocampus is involved in the development of post-ischemic seizures and epilepsy after ischemia (Lei et al., 2014, 2016).

To explore the role of SESN3 in hippocampal hyperexcitability and post-ischemia seizures occurring in diabetic animals, the present study first examined changes in expression of SESN3 and neuronal excitability after ischemia in diabetic animals. Secondly, we employed SESN3 knockout (KO) mice to investigate the casual effect of SESN3 on post-ischemic seizures. Our results showed that SESN3 up-regulation played an important role in hippocampal hyperexcitability and contributed to post-ischemia epileptogenesis in diabetic animals, through down-regulation of Kv4.2-mediated *I*_*A*_ currents.

## Materials and Methods

### Animals

Experimental protocols were approved by the Institutional Animals Care and Use Committee of the Indiana University School of Medicine in accordance with the care and use of laboratory animals. Male mice of C57B16/J (25–30 g, 5–7 weeks old) and male Wistar rats (150–200 g, 5–7 weeks old) were used in the present study. The mice were randomly divided into 4 experimental groups: Control (CTL, *n* = 10); ischemia (IS, *n* = 10); diabetes (STZ, *n* = 10); and diabetes with ischemia (STZ-IS, *n* = 27). SESN3 KO mice (*n* = 7) were provided by Dr. Charlie Dong, Department of Biochemistry and Molecular Biology, Indiana University School of Medicine (Tao et al., 2015). Rats were randomly divided into 4 groups: Control (CTL, *n* = 10); ischemia (IS, *n* = 10); diabetes (STZ, *n* = 10) and diabetes with ischemia (STZ-IS, *n* = 17).

### Diabetic Animal Model

Streptozotocin (STZ)-induced diabetes is a well-established animal model (Malhotra et al., 1981). To induce hyperglycemia, mice were intraperitoneally injected with STZ dissolved in 0.1 M citrate buffer (pH4.5, prepared just before use), final concentration of 7.5 mg/ml. Mice were fasted for 4 h before STZ injection (150 mg/kg) and had access to water freely. Rats were fasted overnight and STZ (50 mg/kg) was injected (i. p.) for two consecutive days. Blood was collected from the saphenous vein. Blood glucose level (BGL) was measured by One Touch Ultra glucometer 6 days after STZ injection. The animals with a BGL of ≥300 mg/dL were considered diabetic (Srinivasan et al., 2005). Transient global ischemia was performed at 1 week after STZ injection.

### Transient Forebrain Ischemia Model

Mice were initially anesthetized with 5% isoflurane in a chamber. After shaving hair around the neck, mice were placed on a surgery board and anesthetized with 2% isoflurane via a nasal mask. Then the bilateral common carotid arteries were separated and clipped for 15 min. During clipping, the rectal temperature was maintained at 37°C with a heating lamp via a feedback system. Then the wound was stitched and the mice injected with buprenorphine SR to relieve the pain.

Transient global ischemia in rats was induced by transient occlusion of bilateral common carotid arteries and permanent occlusion of both vertebral arteries (4-VO) as described in our previous studies (Ruan et al., 2009). On the first day, the adult male Wistar rats were anesthetized with 2% isoflurane via a nasal mask. Both vertebral arteries were electrocauterized. A length of silicone tubing (0.025″ I.D., 0.047″ O.D.) was placed loosely around each common carotid artery and passed through two holes in a small teflon button before being tied in a loop. A suture line was tied at the end of the loop. The incision was closed with wound clips. The rats were then allowed to recover from anesthesia overnight. The next day, the carotid arteries were occluded in the lightly anesthetized rats. The 2% lidocaine-HCl (Sparhawk Laboratories., Lenexa, KS, United States) was applied as a local anesthetic in the region of incision. Under light anesthesia, the ventral neck suture was removed. The silicone tubing was then threaded and drawn through a 2-cm plastic cylinder, compressing the artery against the Teflon button. During occlusion, the isoflurane was removed and the rectal temperature was maintained at 37°C with a heating lamp via a feedback system, and the completeness of global ischemia was confirmed by testing the loss of righting and pupil reflexes. After the termination of 15 min occlusion, the silicone tubing, teflon buttons and sutures were removed and the incision was closed. The rats were injected with buprenorphine SR to relieve the pain. Rats were then allowed to recover in a standard cage.

Behavioral (i.e., convulsive) seizures include symptoms on a scale from III to V using the Racine scale (Racine, 1972). A class III seizure was characterized by forelimb clonus, an erect tail and lordotic posturing. A class IV was characterized by continued forelimb clonus and rearing on hindlimbs. Animals showing all of these behaviors in combination with a fall were defined as having a class V seizure (Lei et al., 2014, 2016).

### Histochemistry Staining and Immunohistochemical Staining

#### HE Staining

Mice were sacrificed immediately after seizure onset. The animals were deeply anesthetized using 5% isoflurane and perfused with phosphate buffered saline (PBS, 0.01 mol/L, pH 7.4) for about 3 min, followed by 4% paraformaldehyde in PBS for about 10 min. After 1 day of post fixation, 40-μm coronal sections containing the hippocampus were cut with a Vibratome (VT1000; Leica, Nussloch, Germany). The sections were stained with hematoxylin and eosin (H&E, Thermo Fisher Scientific, Pittsburgh, PA, United States).

#### Immunohistochemical Staining

Rats were deeply anesthetized, perfused with PBS and fixed with 4% paraformaldehyde in PBS (Lei et al., 2012). After being postfixed overnight, six sets of coronal sections of the hippocampus were cut (40 μm) with a Vibratome (Technical Products International) and collected in PBS. The sections from all groups were stained together in each immunohistochemical step. The sections were incubated for 30 min in 0.3% H_2_O_2_ (Sigma) to quench the endogenous peroxidase activity. Subsequently, the sections were blocked and permeabilized in permeabilization solution (5% goat or horse serum, 0.1% Triton X-100 in PBS) for 1 h at room temperature before they were incubated with rabbit polyclonal anti-SESN3 (1:100; Abcam, Cambridge, MA, United States) in permeabilization solution overnight at 4°C. After being washed, the sections were incubated with biotinylated goat anti-rabbit IgG (1:200; Vector) in blocking solution (5% horse or goat serum in PBS) for 1 h at room temperature. After three washes, the sections were processed with Vectastain ABC Kits (HRP, Vector) and visualized by using 0.05% 3,3’-diaminobenzidine (DAB, sigma) in PBS containing 0.015% H2O2 for 1–2 min. The sections were then mounted onto slides, air dried, dehydrated in graded series of ethanols, infiltrated in xylene, and embedded in paraffin before they were examined with a microscope (BX50; Olympus). Images were acquired with a digital camera coupled to control software (DP70-BSW; Olympus) at 4 and 20 X magnification. The conditions for catching images was controlled constantly throughout all sections.

### Western Blotting

Mice were deeply anesthetized with 5% isoflurane and perfused with cold saline. The brain was removed quickly and the hippocampus was dissected and placed in an EP tube with EDTA-RIPA buffer (Boston BioProducts, Worcester, MA, United States). The tissue was homogenized by smashing and ultrasonication. Insoluble cell debris was removed by centrifugation at 13,000 rpm for 30 min at 4°C and the resulting supernatants were collected for analysis. Protein concentration was determined by BCA assay with a commercial Kit (Bio-Rad, Hercules, CA, United States) and then adjusted to the same concentration among different samples followed by being boiled with 2X sodium dodecyl sulfate gel-loading buffer (Invitrogen, Carlsbad, CA, United States). Then sodium dodecyl sulfate polyacrylamide gel electrophoresis (SDS-PAGE) was carried out. Following being concentrated in 5% stacking gel, equal amount of protein (30 μg) was separated in 10% SDS-PAGE gels and transferred to 0.2 μm PVDF membrane. Then the membrane was blocked in 3% BSA in TBST for 2 h at room temperature (RT) followed by being incubated in primary antibody overnight at 4°C. We used rabbit polyclonal anti-Kv4.2 (1:1000; Chemicon, Temecula, CA, United States), rabbit polyclonal anti-SESN3 (1:1000; Abcam, Cambridge, MA, United States), mouse monoclonal anti-GAPDH (1:10000; Thermo Fisher Scientific, Rockford, IL, United States), or mouse monoclonal anti-β-actin antibodies (1:20000; Sigma). After rinsed in TBST 3 times for 10 min each, the membranes were incubated in the HRP-conjugated secondary antibodies (Vector, Burlingame, CA, United States) for 1 h at room temperature. After incubation, membranes were rinsed three times for 10 min with TBST again. Following a final rinse, the signals were detected with enhanced chemiluminescence (Amersham, Piscataway, NJ, United States) and visualized by exposing to X-ray films (Fuji, Tokyo, Japan). Band densitometry analysis of membrane was performed by using scanned images of non-saturated immunoblot films in NIH Image J 1.37.

### Real-Time RT-PCR

Total RNA was prepared using an Absolutely RNA RT-PCR Miniprep kit (Stratagene, Cedar Creek, TX, United States). Reverse transcription of 1-μg total RNA to cDNA was performed using the StrataScript qPCR cDNA synthesis kit (Stratagene). Real-time quantitative polymerase chain reaction amplification was performed in a Stratagene MX 3005P Thermal Cycler (La Jolla, CA, United States) using RT2 SYBR Green qPCR Master Mix. Primers for SYBR Green-based real-time PCR were purchased from SuperArray Bioscience (Frederick, MD, United States). The relative amount of target mRNA was calculated using the comparative cycle threshold (C_*t*_) method and normalizing each target gene with C_*t*_ of housekeeping gene, GAPDH.

### Electrophysiological Recording

Electrophysiological techniques used in this study have been established in our lab for years (Li et al., 2009; Lei et al., 2012). Rats were be deeply anesthetized with 5% isoflurane and perfused transcardially with cold sucrose solution. The brain was quickly removed and immersed in ice-cold artificial cerebrospinal fluid (ACSF). Brain slices containing hippocampus (300-μm thickness) was cut using a vibratome (Leica VT1000). Hippocampal slices were incubated in ACSF (continuously equilibrated with 95% O_2_-5% CO_2_) for >1 h at room temperature (∼ 24°C) before being transferred to the recording chamber. The ACSF was comprised of the following (in mM): NaCl 130; KCl 3; CaCl2 2; MgCl_2_ 2; NaH_2_PO_4_ 1.25; NaHCO_3_ 26; Glucose 10. Recording electrodes were prepared from borosilicate glass (Warner Instruments, Hamden, CT, United States) using a horizontal electrode puller (Sutter Instruments, Novato, CA, United States) to obtain a tip resistance of 3–5 MΩ. Neurons were visualized with an infrared-DIC microscope (Olympus BX50WI) through a CCD camera. Recordings were made using Axopatch 200B (Axon Instrument). Capacitive transients were reduced by adjusting the capacitance compensation of the amplifier. Signals were filtered at 2 KHz and digitized at a sampling rate of 5 KHz using data acquisition program Axograph 4.6 (Axon Instruments). The data were stored in a Macintosh computer for offline analysis.

Whole-cell current-clamp recording were used to study the membrane properties. The internal solution contained (in mM): KMeSO4 120, KCl 12, MgCl2 1, EGTA 1, CaCl2 0.2, HEPES 10, and Mg-ATP 2, pH 7.3, 290–295 mOsm/L. Depolarizing current pulses (800 ms, 20–180 pA) were applied to evoke either a single firing or repetitive firings. The membrane input resistance (R_*in*_) was derived from the linear portion of the current-voltage curve. The spike latency was measured from the onset of the current injection to the peak of the first action potential. The spike threshold will be measured at the beginning of the upstroke of the first action potential (AP).

To study synaptic transmission, the membrane potential was held at -80 mV. The internal solution contained (in mM): CsCl 43, CsMeSO4 92, TEA 5, EGTA 2, MgCl2 1, HEPES 10, and Mg-ATP 4. For miniature analysis, an episode of 5–10 min was collected for miniature excitatory postsynaptic currents (mEPSCs). Tetrodotoxin (TTX, 1 μM) was applied during mEPSCs recording. Bicuculline (BIC) was used to block GABA_*A*_ receptors at a concentration of 30 μM. The amplitude and frequency of the synaptic events were measured. EPSCs above a threshold (2.5 SDs above baseline noise) was automatically detected by a sliding template algorithm and manually checked off-line.

To study *I*_*A*_ currents, the internal solution was the same as that used for measuring the membrane properties. The voltage-dependent outward potassium currents were evoked by voltage steps (from −90 mV to +60 mV in 10 mV increments, 400 ms) following a 300 ms hyperpolarizing pulse of −130 mV in the presence of TTX (1 μM) and CdCl_2_ (300 μM). *I*_*A*_ currents were isolated by subtracting the currents evoked after depolarized pre-pulses (0 mV, 100 ms) from those evoked without depolarized pre-pulses.

### Statistics

Student *t*-test was used to compare two groups of data. Paired *t*-test was used to compare the same values before and after treatment. The post-ischemia seizure rates in different experimental groups were compared with the Chi-square test. One-way analysis of variance (ANOVA) was used for multiple-group comparisons followed by *post hoc* analysis with Fisher’s PLSD. All statistical analyses were performed via StatView 5.0 software (Abacus Concepts). The difference was considered significant when *P* < 0.05.

## Results

### The Post-ischemic Seizure Incidence Significantly Increased in Diabetic Mice

**FIGURE 1:**
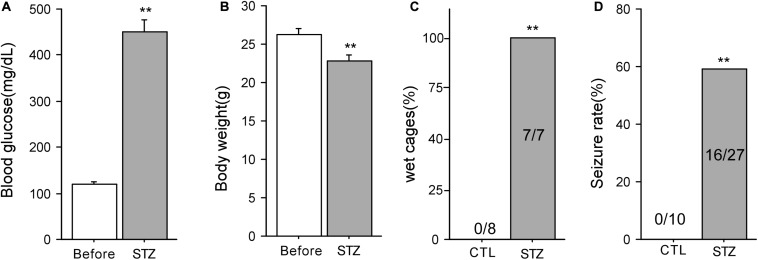
Diabetic animal model in mice and post-ischemic seizures. **(A)** Blood glucose levels of mice before and 7 days after STZ injection (*n* = 14). **(B)** The body weight of mice before and 7 days after STZ injection (*n* = 19). **(C)** The percentage of wet cages holding control and diabetic mice. **(D)** 59% of the mice with diabetes developed seizures following 15-min ischemia, whereas none of the mice with normoglycemia had seizures after ischemia. **P < 0.01 vs. control groups.

Seven days after STZ injection, the blood glucose levels of animals were significantly elevated compared with those before injection (Before: 119.4 ± 5.9 mg/dL; 7 days after: 450.1 ± 25.6 mg/dL, *n* = 14, *P* < 0.01, Figure 1A). After STZ injection, the body weight of these mice decreased markedly (Before: 26.3 ± 0.8 g; 7 days after: 22.8 ± 0.7 g, *n* = 19, *P* < 0.01, Figure 1B). Furthermore, seven cages housing the diabetic mice (100%) were found wet with urine after STZ injection while the cages housing the control mice (0%) were in normal condition (*P* < 0.01, Figure 1C). These data indicate that the mice showed typical diabetic symptoms after STZ injection. Although both the control and diabetic mice were subjected to 15 min ischemia, no seizures were observed in 10 control mice after ischemia whereas 16 out of 27 (59.2%) diabetic mice exhibited post-ischemia seizures (*P* < 0.01, Chi-square test, Figure 1D). The time of first onset of seizures in mice varied from 0.6 to 22.8 h following ischemia (Median = 5.7 ± 2.1 h, *n* = 12). In our preliminary experiment, we found that the frequence of post-ischemia seizures increased and finally developed into status epilepticus (SE) before the mice died within 12 h after the first onset of seizures. SE is a very severe type of seizure, starting with forelimbs and hind limbs rigidly pushed away from the body, followed by violent shaking, vibrating, and jumping (Lei et al., 2014, 2016). Therefore, we sacrificed mice immediately after the first onset of seizures (Supplementary Videos).

### No Apparent Cell Death Is Observed Right After Post-ischemic Seizures in Diabetic Mice

**FIGURE 2:**
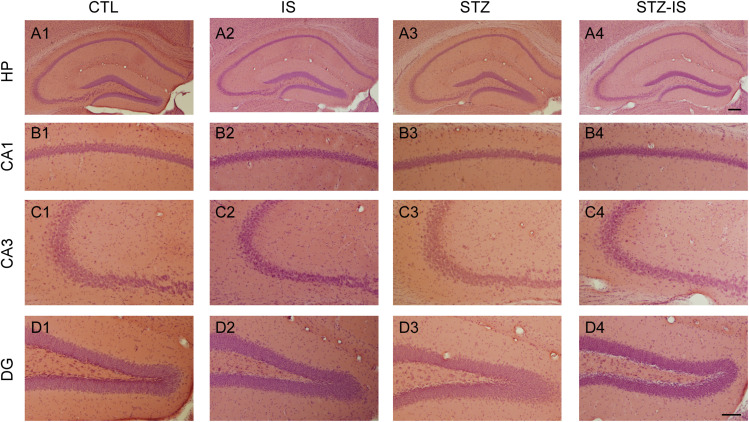
Histology of the hippocampus in different experimental conditions. Representative photographs show H&E staining of brain sections of CA1, CA3, and DG in control (CTL), ischemia (IS), diabetes and diabetes-ischemia (STZ) mice. No apparent cell death was observed in the hippocampus 12 h after ischemia in normoglycemia and diabetic mice. The scale bar of **(A4)** (200 μm) is equal to **(A1–A3)**. And the scale bar of **(D4)** (100 μm) is equal to **(B1–D3)**.

In order to investigate whether the post-stroke seizure in diabetic mice was attributable to neuronal death, we examined the morphology of hippocampal neurons following ischemia using H&E staining (Figure 2). Ischemic neuronal injury is determined by the eosinophilic cytoplasm, nuclear pyknosis, and shrinkage of the cell body in H&E sections. The results showed that there was no obvious neuronal damage in the CA1 area, CA3 area, and dentate gyrus (DG) among the control, ischemic, diabetic, and diabetic-ischemic mice before they were sacrified.

### Higher Expression of SESN3 in Post-ischemia Seizures in Diabetic Animals

**FIGURE 3:**
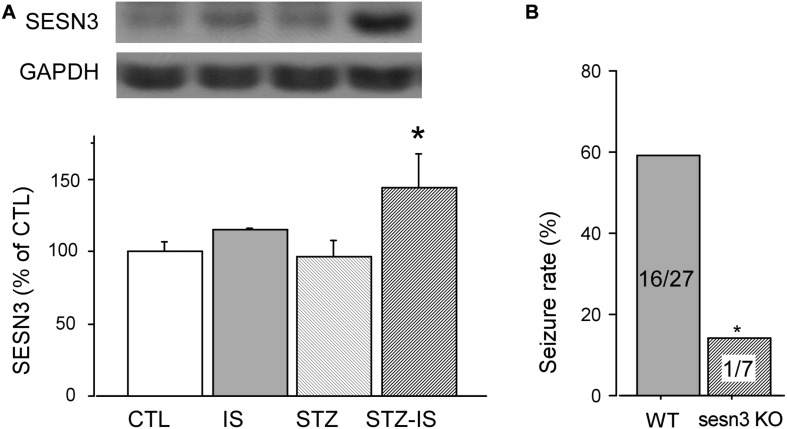
Western blot results showing SESN3 protein levels increase in diabetic mice after 15-min ischemia. **(A)** The expression of SESN3 increased significantly in diabetic ischemia group (*P < 0.05 vs. the control group). **(B)** Compared to wild type diabetic mice following ischemia, the post-ischemic seizure incidence significantly decreased in diabetic sesn3 KO mice, *P < 0.05 vs. the control group.

Sestrin3 positively regulates epilepsy gene network that contains a specialized, highly expressed transcriptional module encoding proconvulsive cytokines and Toll-like receptor signaling genes in human hippocampal epilepsy (Johnson et al., 2015). In order to reveal whether SESN3 is related to post-ischemia seizures or diabetes, we detected the expression of SESN3 in each group of animals. As shown in Figure 3A, compared with the control (100 ± 8.4%), there were no significant changes in the expression of SESN3 protein in the ischemia group (110.7 ± 4.7%), or diabetic group, both *P* > 0.05. However, the expression of SESN3 protein increased significantly in diabetic ischemia group (144.5 ± 23.6% vs. 100 ± 8.4%, *P* < 0.05).

The above results suggest that the expression of SESN3 is related to ischemic diabetes-induced seizures. To prove this, we employed sesns3 KO mice to establish sesns KO ischemic mice. The diabetes of sesn3 KO mice was also produced by injection of STZ. The blood glucose level was similar to the wild type mice which was 120.7 ± 6.1 mg/dL before injection of STZ and reached 455.9 ± 30.8 mg/dL (*n* = 10) 7 days following injection of STZ. After the mice underwent 15 min global ischemia, the seizure incidence was significantly decreased to 14.2% compared to WT ischemic diabetes mice (WT-STZ-IS) (*n* = 7, *P* < 0.05, Figure 3B). The above results demonstrate the critical role of SESN3 in post-ischemia seizures in diabetic mice.

### Abnormal Electrical Activity Was Induced in Post-ischemic Seizures Under Diabetic Conditions

**FIGURE 4:**
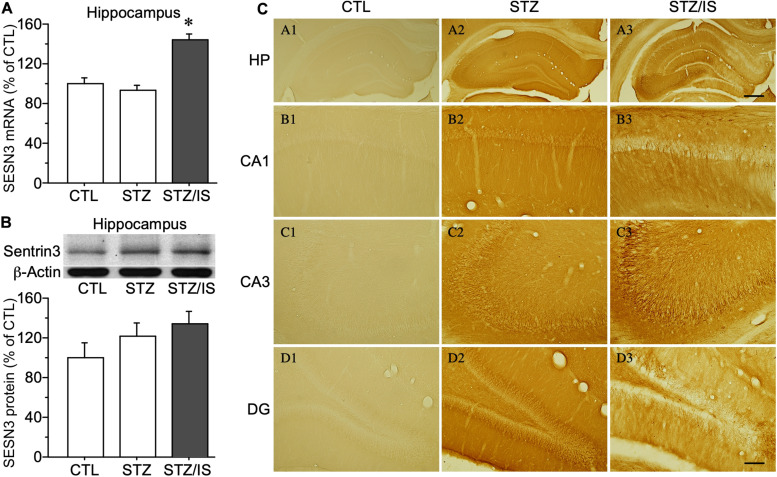
Quantitative PCR and Western blot results showing changes in sesn3 mRNA and protein levels in different experimental groups **(A)** Sesn3 mRNA levels in the hippocampus increased in diabetic rats after 15-min ischemia, *P < 0.05 vs. CTL and STZ groups. **(B)** The SESN3 protein levels in the hippocampus also appeared an increasing trend in diabetic rats after ischemia. **(C)** The intensity of immunostaining of SESN3 in the hippocampus is higher in diabetes after ischemia, especially in the CA3 region **(C3)**. The scale bar of **(A3)** (500 μm) is equal to **(A1–A3)**. And the scale bar of **(D3)** (100 μm) is equal to **(B1–D3)**.

To investigate mechanisms of post-ischemic seizures in diabetic animals, the excitability of hippocampal neurons of rats was examined among different groups. Diabetic rats were induced by injection of streptozotocin (STZ) (50 mg/kg, i.p.) each day for two consecutive days. The blood glucose levels were significantly increased in diabetic rats (422 ± 16 mg/dL, *n* = 10) at 1 week after STZ injection when compared with the controls (117 ± 5 mg/dL, *n* = 10), *P* < 0.01. The post-ischemia seizure rate was significantly increased in diabetic rats as compared with the controls (100% in diabetes vs. 0% in control, *P* < 0.05). To reveal the changes in SESN3 expression of diabetic animals after ischemia, the mRNA and protein levels of SESN3 were compared in different experimental groups. Diabetic rats were sacrificed shortly after the onset of seizures following ischemia. As shown in Figure 4A, quantitative PCR data indicated that SESN3 mRNA expression was 43.8% higher in diabetic animals after ischemia than that of the controls (STZ-ischemia: 143.8 ± 6.0% of control, *P* < 0.05). Western blotting data showed that SESN3 protein in hippocampus increased by 21.3% in diabetic rats and by 34.4% in diabetic rats after ischemia when compared with the controls (Figure 4B). Immunohistochemical studies showed that intensity of SESN3 staining in the hippocampus was increased in diabetic rats and it was strongest in the CA3 region of the hippocampus in diabetic-ischemic rats (Figure 4C).

**FIGURE 5:**
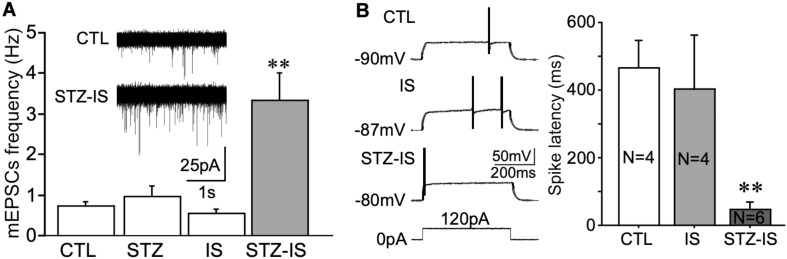
Electrophysiological recording displaying changes in neuronal excitability in different experimental groups. **(A)** The frequency of mEPSCs in granule cells significantly increases after ischemia in diabetic rats (**P < 0.01 vs. CTL). **(B)** The latency of the first spike evoked by depolarizing current pulse in granule cells of STZ/IS animals was significantly shorter than the control ones (**P < 0.01), suggesting that the membrane excitability was increased after ischemia in diabetic rats.

Whole-cell recording of dentate granule cells of diabetic rats was performed. The animals were sacrificed immediately after onset seizure and transverse brain slices at 300 μm were prepared. mEPSCs of dentate granule cells were compared in each groups. As shown in Figure 5A, in comparison with the control rats, the frequency of mEPSCs was significantly increased in diabetic animals after ischemia (control: 0.74 ± 0.10 Hz, *n* = 9 vs. STZ-ischemia: 3.35 ± 0.68 Hz, *n* = 7, *P* < 0.01). Furthermore, the membrane properties of these neurons were investigated (Tables 1, 2). The resting membrane potential was depolarized in diabetic animals with ischemia (Control: −89. 3 ± 0.9 mV; STZ-ischemia: −77.2 ± 4.0 mV, *P* < 0.05), the input resistance was also increased (Control: 248.1 ± 37.8 MΩ; STZ-ischemia: 338.5 ± 38.0 MΩ, *P* < 0.05). The latency of first spike was significantly shortened (Figure 5B, Control: 0.467 ± 0.079 s; STZ-ischemia: 0.046 ± 0.024 s, *P* < 0.01). These data suggest that the hyperexcitability of granule cells after ischemia may be related to the onset of seizure.

**TABLE 1:** Passive membrane properties of granule cells after ischemia in diabetic rats.

	Control (*n* = 4)	Ischemia (*n* = 4)	STZ (*n* = 4)	STZ-ischemia (*n* = 6)
RMP (mV)	−89.3 ± 0.9	−84.8 ± 3.8	−79.9 ± 3.6	−77.2 ± 4.0*
*R*_*in*_ (MΩ)	248.1 ± 37.8	284.8 ± 10.5	305.8 ± 19.9	338.5 ± 38.0*

Values are means ± SE, with number of neurons in parentheses. Input resistant (*R*in) is derived from the linear portion of the current-voltage curve (0–0.5 nA). RMP, resting membrane potential. *P < 0.05, *P < 0.01.

**TABLE 2:** Active membrane properties of granule cells after ischemia in diabetic rats.

	Control (*n* = 4)	Ischemia (*n* = 4)	STZ (*n* = 4)	STZ-ischemia (*n* = 6)
SpkH (mV)	98.4 ± 4.6	91.1 ± 5.0	109.0 ± 3.8	85.8 ± 6.9
SpkL (ms)	0.467 ± 0.08	0.404 ± 0.16	0.321 ± 0.07	0.046 ± 0.02**
SpkD (ms)	0.852 ± 0.08	0.883 ± 0.03	0.643 ± 0.03	0.666 ± 0.04*
SpkT (mV)	−41.8 ± 1.3	−36.3 ± 1.8	−42.1 ± 0.6	−32.8 ± 2.3*
Rheobase (nA)	110.0 ± 17.3	115.0 ± 12.6	90.0 ± 5.8	170.0 ± 23.5*

Values are means ± SE, with number of neurons in parentheses. SpkH, spike height is the peak absolute voltage value, measured from the resting membrane potential. SpkL, the latency of first spike, measured from the onset of the current injection to the peak of the first action potential. SpkD, spike width is the width at half-maximal spike amplitude, measured at half of the peak amplitude of the action potential. SpkT, spike threshold is the voltage at which action potential is initiated, measured at the beginning of the upstroke of the first action potential. Rheobase is determined as the minimal intensity of depolarizing current pulse to trigger one action potential during 1 s. *P < 0.05; **P < 0.01.

**FIGURE 6:**
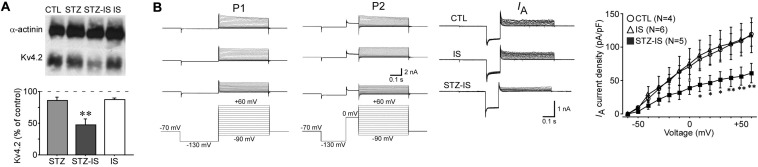
Electrophysiological recording showing changes in neuronal inhibition in different experimental groups. **(A)** Western blotting showing a reduction of total Kv4.2 protein levels in seizure rats after 15-min diabetic ischemia (**P < 0.01 vs. control group). **(B)** *I*_*A*_ currents in granule cells were reduced in diabetic rats after ischemia (*P < 0.05 vs. control group).

*I*_*A*_ currents play an important role in controlling neuronal excitability and therefore may contribute to seizure generation after ischemia. Kv4.2 is the major subunit of *I*_*A*_ channels. Western blotting showed a significant reduction of total Kv4.2 protein levels in the hippocampus of seizure animals (47.1 ± 9.7% of control, *n* = 3, *P* < 0.01; Figure 6A). *I*_*A*_ was recorded from dentate granule cells of hippocampal slices. The current density of *I*_*A*_ was reduced after 15-min ischemia in diabetic animals (Figure 6B). These data suggest that *I*_*A*_ currents of granule cells in diabetic rats are decreased after ischemia and contribute to the increase of excitability.

## Discussion

Epilepsy has long-term devastating effects on stroke victims and a financial burden on society. It is well known that diabetic patients have a higher incidence of stroke and worse post-stroke seizures as compared to the non-diabetic patients (Krakow et al., 2010; Adelow et al., 2011). However, the mechanisms of post-stroke seizure/epilepsy in patients with diabetes are not fully understood and no effective treatment is currently available to those patients. The current treatment of seizures following brain injury is symptomatic and could not prevent the recurrent epilepsy (Temkin et al., 1990; Temkin, 2009).

Our studies and others have shown that transient global ischemia induces selective neuronal death in vulnerable area in the CA1 pyramidal neurons of the hippocampus, cortical pyramidal neurons and spiny neurons of the striatum, rather than causes infarct area of the cerebral cortex (Kirino, 1982; Pulsinelli et al., 1982; Ruan et al., 2006; Shi et al., 2017). Delayed neuronal death usually occurs in the hippocampus at 2–3 days after transient global ischemia. That is why we did not detect the cell death in the hippocampus at 12 h after ischemia. In this case, the incidence of seizures after ischemia seems not related to the CA1 neural death of the hippocampus.

Recent studies found that sestrin 3 is a regulator of a proconvulsant gene network in human epileptic hippocampus and seizures are reduced by inhibition of high rates of glucose metabolism via lactate dehydrogenase (LDH) (Johnson et al., 2015; Sada et al., 2015). SESN3 has been implicated in the regulation of multiple pathways including AMPK, mTORC1, and mTORC2 axes, which in turn regulates hepatic insulin signal transduction and glucose metabolism (Tao et al., 2015). Therefore, it is conceivable that SESN3 is associated with the seizures following ischemia in diabetes.

To investigate the role of SESN3 in post-ischemia seizures under diabetic condition, we established a mouse model of post-ischemia seizures. Compared with the rat model we used before (Xia et al., 2016), it is useful to use knockout mice to establish the causal relationship of SESN3 in post-ischemic seizures. First, we executed STZ injection to induce diabetes in mice. At 7 days after injection, the increased blood glucose, the reduced body weight and the enhanced amount of urine indicate the diabetic status. The present study showed that there was a trend of increased SESN3 protein level and a significant increase in SESN3 mRNA level in STZ-IS group, which is positive associated with post-ischemic seizures. This suggests that SESN3 gene plays important role in post-ischemic seizures.

Global cerebral ischemia occurs when the blood supply to the entire brain or a large part of the brain is disrupted, resulting in the tissue deprived of oxygen and glucose that damages the brain. It is well known that diabetes exacerbates ischemic brain damage (Tureyen et al., 2011; Wei et al., 2013). We examined the brain damage in our mouse model. It is well known that transient global ischemia mainly damages the CA1 pyramidal neurons of hippocampus rather than the CA3 neurons, and this kind of cell death was delayed until 3 days after ischemia and reperfusion (Kirino, 1982; Arai et al., 1986). In the present study, our HE staining showed that there was no obvious cell death both in CA1 and CA3 area of hippocampus less than 24 h after ischemia in wild type mice with normal blood glucose, which was consistent with the delayed neuronal death. We also did not observe neuronal death in the hippocampus when the mouse brains were immediately fixed at the onset of post-ischemic seizures in diabetic animals. However, if the mouse brains were fixed after the occurrence of seizures, the CA1 neurons remained intact while some neuronal death was observed in the CA3 and hilus areas (Lei et al., 2014, 2016). The above observation suggests that the neuronal death in the CA3 and hilus areas may not be a result of ischemia, but may be related to the seizure activities.

Increase of neuronal excitability is a widely recognized cause of seizures/epilepsy generation. Hippocampus is a critical structure as an epileptic focus and epileptogenic zone, and thus as a target of resections for surgical treatment of temporal lobe epilepsy. It is possible that the both membrane excitability and excitatory synaptic transmission are enhanced in hippocampal neurons in diabetic animals after ischemia, which results in seizures and epilepsy. A long-term hyperexcitability and seizure-like activities have been recorded in cortical and hippocampal slices prepared from animals after global ischemia (Luhmann and Heinemann, 1992; Congar et al., 1995). Recently, combining *in vivo* EEG and *in vitro* voltage-clamp recording, Epsztein et al. have shown that the seizure threshold in CA3 pyramidal neurons is reduced after cerebral ischemia (Epsztein et al., 2006). Intrinsic membrane properties play an important role in neuronal excitability and therefore the alteration of membrane properties after ischemia is closely associated with seizure generation. On the other hand, Congar et al. (1995) have shown that the resting membrane potential of CA3 neurons shift to more positive levels after ischemia. In addition, in epileptic animals, the inhibitory basket cells in dentate gyrus receive less excitatory synaptic drive, the transmission failure to granule cells is increased (Lowery et al., 2009). Consistent with the above observations, the frequency of miniature inhibitory postsynaptic currents (mIPSCs) in granule cells is reduced in TLE or kainate induced seizure (Shao and Dudek, 2005). As we know there is no evidence showing glia cells express SESN3 by far. Therefore, it is possible that the both membrane excitability and excitatory synaptic transmission are enhanced in hippocampal neurons in diabetic animals after ischemia, which results in seizures and epilepsy.

In recent years, accumulating evidence suggests the involvement of inflammatory and immune processes in the etiopathogenesis of seizures (Vezzani and Granata, 2005). Inflammatory responses induced by stroke are associated with acute symptomatic seizures and a high risk of developing epilepsy (Bartfai et al., 2007; Vezzani et al., 2011). It is conceivable that up-regulation of sestrins in hippocampus could lead to seizure generation in diabetes after ischemia. However, how sestrins regulate inflammation in the brain and their role in epileptogenesis in diabetes are not clear. Recent studies have shown that glucose regulates the mTOR pathway through a Sestrin-dependent pathway (Ben-Sahra et al., 2013). Several preclinical and some clinical studies have indicated that the hyperactivation of the mTOR signaling pathway appears involved in acquiring epilepsy (Griffith and Wong, 2018). Here, we found an increased neuronal excitability, together with decreased *I*_*A*_ currents and Kv4.2 in the hippocampus. It is well-known that *I*_*A*_ currents are important gatekeepers of excitability and synchronicity in the CNS. Somadendritic *I*_*A*_ currents are mainly mediated by the potassium channel Kv4.2. *I*_*A*_ currents and Kv4.2 protein levels are decreased in rodent models of epilepsy, suggesting that the downregulation of *I*_*A*_ currents is a pathological mechanism in epilepsy (Lei et al., 2012, 2014). So far, the mechanisms that lead to this downregulation of *I*_*A*_ currents and Kv4.2 protein levels are still unclear. activation of mTORC1 signaling increases the abundance of sestrins which prevents ROS accumulation via inhibition of mTOR (Lee et al., 2010). It has been reported that mTOR activity was increased in animals with epilepsy (Ostendorf and Wong, 2015; Citraro et al., 2016). Evidence showed that mTOR dysregulation could potentially contribute to hyperexcitability and recurrent seizures by regulating Kv4.2-mediated *I*_*A*_ currents (Yao et al., 2013; Nguyen and Anderson, 2018). We speculate that the SESN3-regulated mTOR pathway might cause the downregulation of Kv4.2 and *I*_*A*_ currents, resulting in neuronal hyperexcitability and the development of post-ischemic seizures under diabetic condition.

We found that the frequency of mEPSCs was significantly increased in diabetic animals after ischemia, and no significant changes was observed in the amplitude of mEPSCs. This finding suggest that presynaptic changes may alter firing properties of postsynaptic DG cells. NMDA receptors are assembled from the NR1 subunit and at least one type of NR2 subunit, mainly NR2A and/or NR2B. NR2A-containing NMDA receptors are believed to be located predominantly at synaptic sites, whereas NR2B-containing NMDA receptors are located predominantly extrasynaptically. Distinct roles of NR2A- versus NR2B-containing or synaptic versus extrasynaptic NMDA receptors have been extensively studied in synaptic plasticity and neurodegenerative diseases (Hardingham et al., 2002). Our previous study have shown that NR2B-containing NMDA receptor-mediated downregulation of Kv4.2 potassium channels (Lei et al., 2010).

Dentate granule cells in the hippocampus survive after ischemic insult but are implicated in seizure generation (Pun et al., 2012). The abnormal hyperexcitability of granule cells results in seizure activities (Young et al., 2009). Our results suggest that the hyperexcitability of granule cells after ischemia in diabetes may be related to the onset of seizure by downregulation of *I*_*A*_ currents.

In summary, the present study demonstrates that seizure incidence increased in diabetic animals after ischemia together with an up-regulation of SESN3 expression. The seizure rate of sesn3 KO mice was significantly reduced after ischemia compared with the wild type mice. The excitability of hippocampal neurons was increased and Kv4.2-mediated *I*_*A*_ currents was decreased in these animals. These results suggest that up-regulation of SESN3 contributes to post-ischemia seizures in the diabetic condition.

However, there are some shortcomings of the study, such as due to time point of sacrifice changes can be cause or consequence of seizures, lack of non-diabetic sesn3 KO group, and lack of EEG for quantification of seizure activity. More precise experimental design is necessary for the further study.

## Data Availability Statement

The raw data supporting the conclusions of this article will be made available by the authors, without undue reservation.

## Ethics Statement

The animal study was reviewed and approved by the Institutional Animals Care and Use Committee of the Indiana University School of Medicine.

## Author Contributions

ZX and YR conceived and designed the experiments. ZS and ZL performed the experiments. ZS, ZL, FW, and LX analyzed the data. ZS, ZL, ZX, and YR wrote, revised, and commented on the manuscript. All authors contributed to the article and approved the submitted version.

## Conflict of Interest

The authors declare that the research was conducted in the absence of any commercial or financial relationships that could be construed as a potential conflict of interest.
