# Supplementary figures and images for: Increased Sestrin3 Contributes to Post-ischemic Seizures in the Diabetic Condition
Source: Front Neurosci. 2021 Jan 15;14:591207. doi: 10.3389/fnins.2020.591207 (PMC7843462; doi:10.3389/fnins.2020.591207)

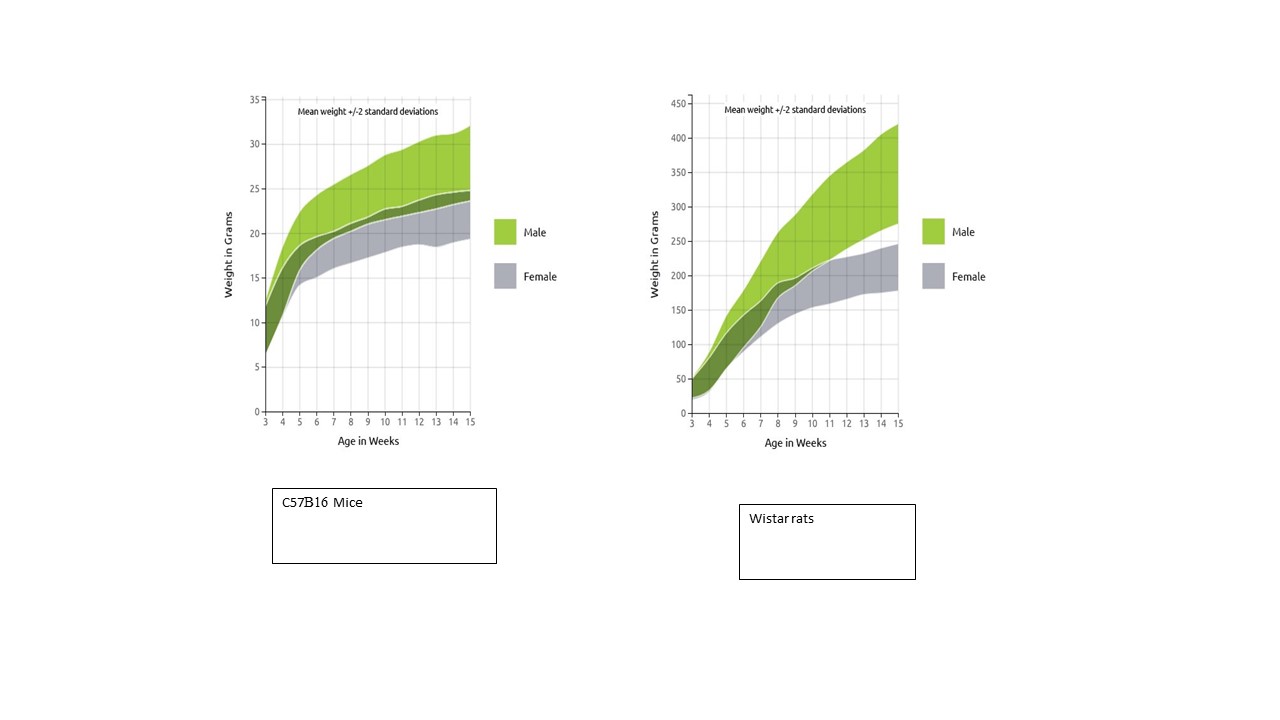

Supplement: Supplementary file 1 [file Image_1.JPEG]
